# Emerging Roles of Non-Coding RNAs in Childhood Asthma

**DOI:** 10.3389/fphar.2022.856104

**Published:** 2022-05-17

**Authors:** Juan Liang, Xiao-Hua Liu, Xue-Mei Chen, Xiu-Ling Song, Wen Li, Yuge Huang

**Affiliations:** ^1^ Department of Pediatrics, The Affiliated Hospital of Guangdong Medical University, Zhanjiang, China; ^2^ Graduate School of Guangdong Medical University, Zhanjiang, China

**Keywords:** childhood asthma, ncRNAs, lncRNAs, miRNAs, circRNAs

## Abstract

Asthma is a chronic airway inflammatory disease in children characterized by airway inflammation, airway hyperresponsiveness and airway remodeling. Childhood asthma is usually associated with allergy and atopy, unlike adult asthma, which is commonly associated with obesity, smoking, etc. The pathogenesis and diagnosis of childhood asthma also remains more challenging than adult asthma, such as many diseases showing similar symptoms may coexist and be confused with asthma. In terms of the treatment, although most childhood asthma can potentially be self-managed and controlled with drugs, approximately 5–10% of children suffer from severe uncontrolled asthma, which carries significant health and socioeconomic burdens. Therefore, it is necessary to explore the pathogenesis of childhood asthma from a new perspective. Studies have revealed that non-coding RNAs (ncRNAs) are involved in the regulation of respiratory diseases. In addition, altered expression of ncRNAs in blood, and in condensate of sputum or exhalation affects the progression of asthma *via* regulating immune response. In this review, we outline the regulation and pathogenesis of asthma and summarize the role of ncRNAs in childhood asthma. We also hold promise that ncRNAs may be used for the development of biomarkers and support a new therapeutic strategy for childhood asthma.

## Introduction

Asthma is one of the most common chronic inflammatory disease characterized by high heterogeneity in pathogenesis (Koczulla et al., 2017; Papi et al., 2018), with symptoms including showing paroxysmal, reversible wheezing, shortness of breath, chest tightness and cough, which occur or intensify at night and/or in the early morning (Holgate et al., 2015). With the exposure of allergens and the use of antibiotics in the first year of infant life, the prevalence of asthma in children is rising (Metsälä et al., 2015). According to the data from the Centers for Disease Control and Prevention (CDC) in 2016, the prevalence of asthma in children aged 5–11 and 12–17 is respectively 9.6% and 10.5% (Haktanir Abul and Phipatanakul, 2019).

**FIGURE 1:**
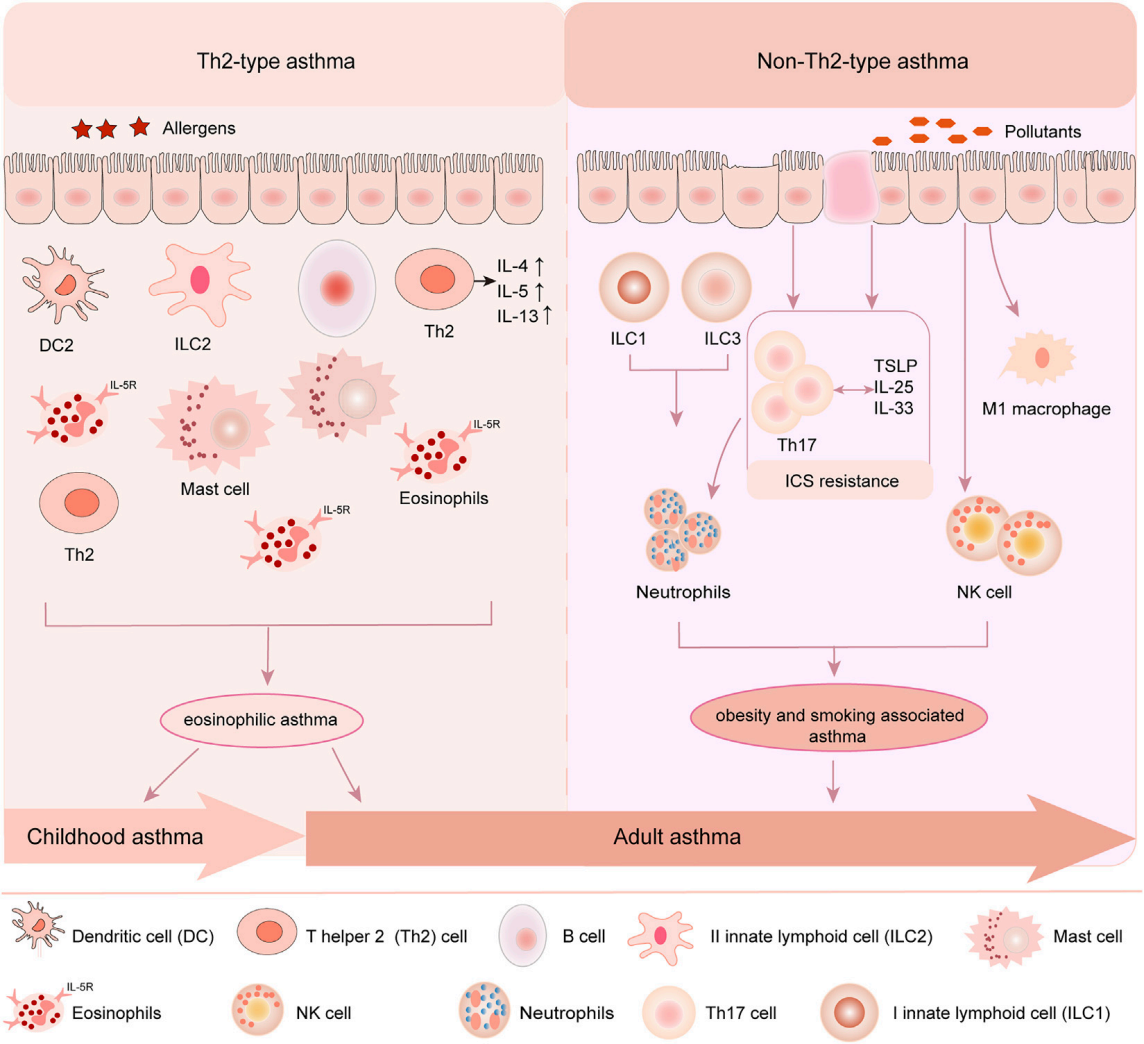
Childhood asthma and adult asthma phenotypes. Childhood asthma and adult asthma are crossed and different in phenotypes. In children, Th2-type asthma is the main common type. Allergen stimulates the recruitment of inflammatory cells such as eosinophils, the proliferation and activation of immune cells such as mast cells and DCs, and induces the injury of airway epithelial cells, which lead to the release of inflammatory factors and Th2 cytokines, such as IL4, IL5 and IL13. In adult, both Th2-type and non-Th2-type asthma are two common types. Upon pollutants stimulation, type I innate lymphoid cells (ILC1) and type III innate lymphoid cells activation (ILC3) activate neutrophils and airway epithelial cells to drive the proliferation of Th17 cells which mediates in turn neutrophil recruitment. Pollutants also contribute to M1 macrophage and NK cell recruitment to the airways, resulting in non-Th2-type asthma in adult.

Asthma affects more than nearly 339 million people globally from childhood to old age, among which childhood asthma is more complicated than adult asthma with multiple phenotypes and variable natural course (Lambrecht and Hammad, 2015; Jat and Kabra, 2017; Qiu et al., 2019; El-Husseini et al., 2020). Evidences confirm that childhood asthma is associated with allergy and strongly driven by genetic and environmental factors which determine the susceptibility and severity of asthma (Lee et al., 2017; Tang et al., 2018a). The typical feature of childhood asthma is airway hyper-responsiveness (AHR), Th2-driven eosinophilic airway inflammation and airway remodeling (Qiu et al., 2019; Li et al., 2021). As for adult asthma, it not only shares the same features with childhood asthma, but also is strongly associated with smoking, obesity, and occupational exposures (Kirenga et al., 2020), which mainly refers to non-Th2-type asthma. Different from adult asthma, childhood asthma is usually related to environmental allergens, such as IgE-dependent Th2-type allergic reaction and viral infections and so on (Ferreira et al., 2019; Qiu et al., 2019; Hammad and Lambrecht, 2021). The onset of childhood asthma involves eosinophils, mast cells, T lymphocytes, neutrophils, airway epithelial cells and their cellular components, leading to increased airway responsiveness, and ultimately resulting in widespread and variable reversible airflow limitation (Foster et al., 2017) (Figure 1).

Airway remodeling mainly refers to a constellation of structural changes induced by asthma, including epithelial injury, increased thickness of the basement membrane, airway angiogenesis and so on (Enomoto et al., 2009; Inoue et al., 2020). Additionally, studies confirm that vascular endothelial growth factor (VEGF), an important mediator in airway structural changes, has a proliferation-inducing effect on normal bronchial epithelial cells and bronchial smooth muscle cells and is increased in children with recurrent early wheezing (Yoshisue et al., 2007; Enomoto et al., 2009). The upregulation of VEGF and downregulation of lymphocyte lead to the development of airway remodeling in asthma (Altman et al., 2019). At present, asthma predictive index is used to judge childhood asthma with wheezing combined with clinical experience at home and abroad, but the diagnosis of asthma in children under 6 years old is still a challenging clinical problem (Lee et al., 2019a; Driscoll et al., 2020). Underdiagnosis of asthma leads to delays in the optimal timing of asthma treatment and may prompt the transition from mild asthma to severe, refractory asthma (Shi et al., 2020). Although the efficacy of pharmacotherapies including inhaled corticosteroids and leukotriene receptor antagonists may help prevent airway remodeling, they cannot reverse the established airway remodeling (Huo et al., 2021). Consequently, it is necessary to strengthen the understanding of the pathogenesis of childhood asthma in order to improve early diagnosis rate. Meanwhile, intervention in the occurrence of airway remodeling is crucial to preventing and treating asthma in children.

NcRNAs are non-protein-coding RNAs molecules, mainly including microRNAs (miRNAs), long non-coding RNAs (lncRNAs), and circular RNAs (circRNAs), which are profoundly involved in post-transcriptional gene expression and participate in the regulation of various biological processes (Defnet et al., 2019; Zangouei et al., 2020; Wang et al., 2021). Some studies have confirmed that ncRNAs play a crucial role in the pathogenesis and regulation of asthma including childhood asthma and adult asthma (Qiu et al., 2018; Yang et al., 2022). Correctly, miRNAs are involved in the regulation of airway inflammation and airway smooth muscle proliferation. LncRNAs not only regulate airway inflammation and airway remodeling, but also affect the regulation of immune responses (Narożna et al., 2017; Elnady et al., 2020; Dai et al., 2021). Moreover, emerging evidence implicates that circRNAs are also involved in the proliferation of smooth muscles and airway remodeling in the progression of asthma (Jiang et al., 2021; Wasti et al., 2021). Hence, ncRNAs are considered as potential biomarkers and promising therapeutic targets for childhood asthma (Narożna et al., 2017; Specjalski and Jassem, 2019).

Clinically, children are special categories of patients and childhood asthma can be misdiagnosed as other diseases with similar symptoms (Abdullahi et al., 2016), which makes the diagnosis of asthma difficult. Besides, even if most childhood asthma patients are relieved after treatment in the early stages according to the international recommendations of the Global Asthma Initiative, drug failure and drug resistance often occur during asthma treatment. That’s to say, up to 5%–10% of children have severe asthma or poor asthma control (Moral et al., 2021; Ntontsi et al., 2021). Therefore, further understanding of the pathogenesis of asthma, especially the identification of differential pathogenesis of asthma between children and adults, will help to find the way of more effective diagnoses and treatments. Accordingly, mechanism study of ncRNAs on the pathogenesis of childhood asthma is worthy of attention. In this review, we mainly demonstrate the pathogenesis of childhood asthma and emerge roles of ncRNAs in asthma. Besides, we hold great promise for the discovery of new ncRNAs biomarkers and therapeutics for asthma.

## Asthma-Associated Pathogenesis

### Immune Factors in the Development and Regulation of Childhood Asthma

Asthma is a heterogenous disease with complex pathogenesis and various phenotypes which can be reclassified *via* molecular biomarkers called “endotypes” (Bond et al., 2018; Assaf and Hanania, 2019). At present, asthma endotypes are divided into T-helper-2(Th2)-high (eosinophilic) and Th2-low (non-eosinophilic). The ratio tilt of T lymphocyte subsets (Th1/Th2) is the most important pathogenesis of asthma (Tang et al., 2018b). Th2-high asthma is related to adaptive immunity and allergic asthma (Lampalo et al., 2019; Song et al., 2019). Upon allergens stimulation, dendritic cells (DCs) activate T-helper-2 (Th2) cells to secrete Th2 cytokines such as IL-4, IL-5 and IL-13 which act on airway epithelial cells, eosinophils, B lymphocytes and other inflammatory cells. This drives B cells to produce a large number of IgE that cross-linking causes degranulation of mast cells to produce a series of inflammatory mediators such as leukotrienes, endothelin, prostaglandin and thromboxane A2, etc. and eventually induce rapid onset (increased IgE) allergies and eosinophilic airway inflammation (Bégin and Nadeau, 2014; Chogtu et al., 2016; McCracken et al., 2016). Studies have shown that childhood asthma is associated with eosinophils in the airways, allergic sensitization and adaptive immunity (Kim et al., 2010). Th2 cells concretely activated by allergens *via* secreting Th2 cytokines IL-4, IL-5 and IL-13, amplify type II inflammation, while T helper 1 (Th1) cells by secreting Th1 cytokines such as IFN-γ, IL-2, lymphotoxin (LT)-α and tumor necrosis factor (TNF)-α and so on, limit type II inflammation and mediate type I inflammation (Foster et al., 2017; Mukherjee and Nair, 2018), which causing childhood asthma. Consequently, the imbalance of the ratio of T lymphocyte subsets (Th1/Th2) is the key mechanism of childhood asthma, whether it is innate immunity or adaptive immunity.

However, non-Th2-type asthma related to non-allergic asthma, which is characterized by emotion, obesity, environmental factors, such as air pollution including ozone, cigarette smoke and so on, may release cytokines such as Il-17 and IFN-γ by activation of Th1 cells, leading to neutrophilic inflammation, M1 macrophage, NKT cell recruitment to the airways and AHRs (Castan et al., 2020; Agache et al., 2021). Nevertheless, non-Th2-type asthma characterized by type I innate lymphoid cells (ILC1) and type III innate lymphoid cells activation (ILC3) is most common in adult asthma. ILC1 in asthma patients promoted eosinophil apoptosis and inhibited eosinophilic airway inflammation (Barnig et al., 2013; Barnig and Levy, 2015). ILC3 produced IL-17A and caused obesity-related AHR effects (Kim et al., 2014). Furthermore, other cytokines (such as IL-17, IL-21, IL-22) also accelerated mucus secretion of airway smooth muscle cells, increased the production of cytokines and chemokines, and promoted neutrophil recruitment by inducing CXC chemokines (Lejeune et al., 2020). In addition, IL-17 promoted airway remodeling by increasing the production of fibrotic cytokines, angiogenic factors, proteases and collagen.

**FIGURE 2:**
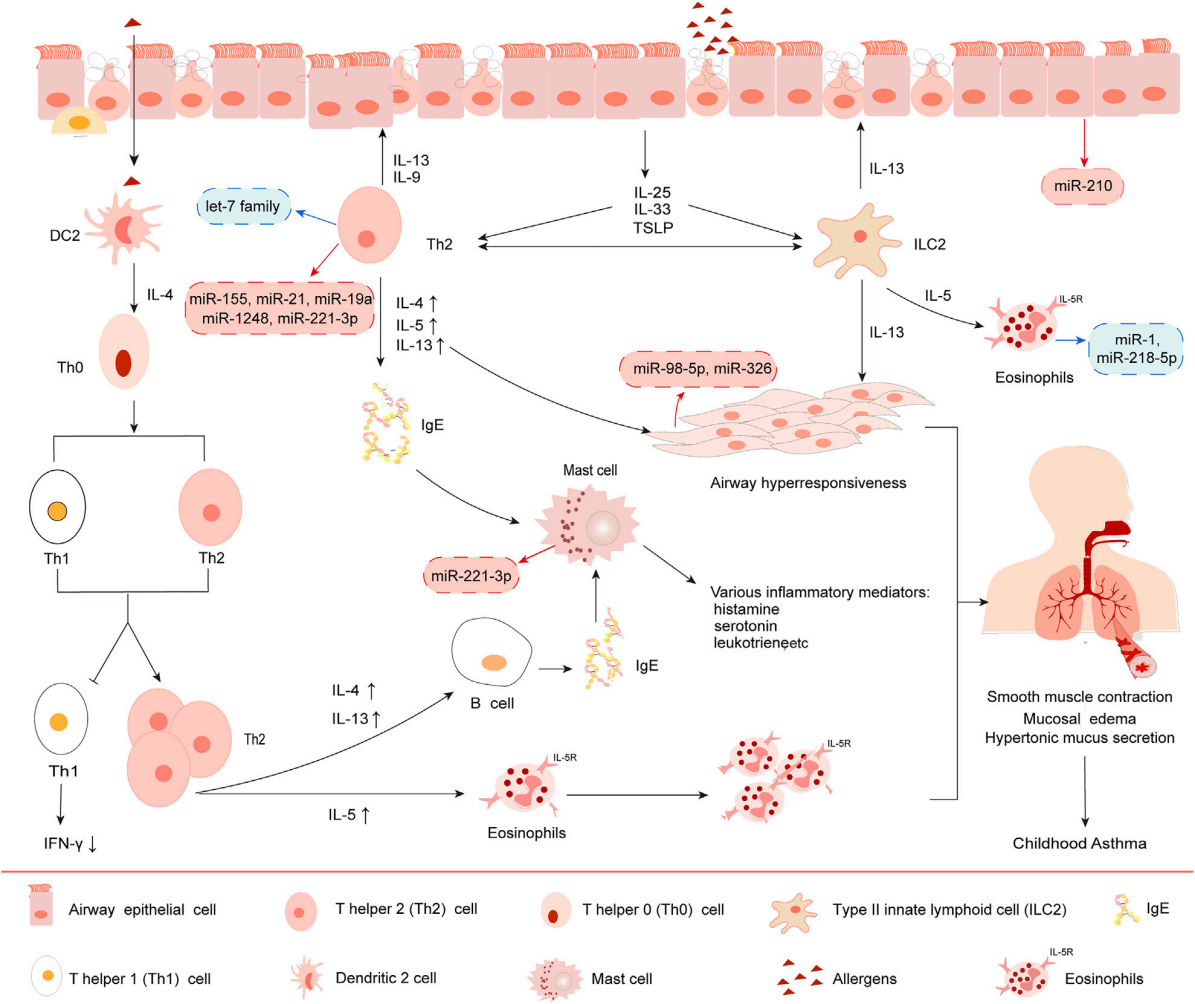
Immunopathogenesis of Th2-type asthma. 1) Under IL-4 induction, dendritic 2 (DC2) cell promotes the development of Th0 cells to Th2, resulting in Th1 (decreased secretion)/Th2 (increased secretion) cell dysfunction. 2) Upon stimulation by allergens, bronchial epithelial cells release IL-25, IL-33 and TSLP, which in turn activates group II innate lymphoid cells (ILC2) and Th2 cells. 3) Subsequently, Th2 cells release Th2 cytokines such as IL-4, IL-5 and IL-13, rather than Th1 cells produce IFN-γ and TNF-β, causing a balanced skewed Th2 cellular immune response. 4) IgE eventually induces rapid onset allergy and cytokines release induced by eosinophils, mast cells and other immune cells, leading to airway inflammation. 5) Moreover, different miRNAs have different effects on the above processes. The red boxes in the picture show typical miRNAs that exert upregulated function in asthma. The blue boxes show miRNAs that exert downregulated function.

Moreover, airway epithelial cells and type II innate lymphoid cells (ILC2) are also involved in adaptive immune. After allergens exposure, epithelial cells polarize macrophages, DC cells, T cells, etc. (Frati et al., 2018; Lejeune et al., 2020), releasing not only IL-4, IL-5, IL-13 and other cytokines but also pro-inflammatory cytokines such as IL-25, IL-33 and thymic stromal lymphopoietin (TSLP) activating ILC2 rather than IFN-γ and TNF-β produced by Th1 cells (Lejeune et al., 2020), which causes Th0 cells polarized into Th2 cells in asthma-specific cytokine environments, resulting in a balanced skewed Th2 cellular immune response. The immune response of Th2 cells further induced changes in the pathophysiological characteristics of asthma, including eosinophils mobilizing IgE, secreting excessive mucus, smooth muscle proliferation and airflow obstruction. Additionally, under the effect of IL-5, eosinophils entered the respiratory tract and triggered a second inflammatory response (Deschildre et al., 2017; Gao et al., 2017). Studies have shown that CD4^+^ T cells are essential for inducing allergic airway disease in newborns, and ILC2s are very important in the pathogenesis of allergic airway disease in adults. Mechanistically, CD4^+^ T cells and ILC2s regulate asthma by promoting the production of IL-13 amplifying type II inflammation (Saglani et al., 2018) (Figure 2). Therefore, understanding the role of immune factors in the development and regulation of asthma will provide opportunities for asthma treatment.

### Mitogen-Activated Protein Kinase Signaling Pathways Involved in Childhood Asthma

Neural signaling pathway also has appealing potential as an application to study pathways to childhood asthma development. Molecular studies have indicated that Mitogen-activated protein kinase (MAPK) pathways, including extracellular signal-regulated kinase 1/2 (ERK1/2), p38 mitogen-activated protein kinase and c-Jun NH2-terminal kinase (JNK), are involved in the inflammatory response and development of airway remodeling during childhood asthma (Lee et al., 2019b; Jia et al., 2019). Concretely, ERK1/2 is involved in airway remodeling (Defnet et al., 2019), while p38 MAPK and JNK are considered as anti-inflammation targets to regulate inflammatory processes *via* phosphorylation of downstream mediators in childhood asthma (Kim and Choi, 2010; Pulido and Lang, 2019; Theodorou et al., 2022). However, literatures about the pathogenesis of MAPK pathways in childhood asthma are limited. In this review we mainly focus on the roles of MAPK signaling pathways in the pathogenesis and related treatment of childhood asthma.

Experiment shows ERK1/2 signaling mainly acts on airway epithelial cells and smooth muscle cells of asthmatic mice (Liu et al., 2008). Concretely, ERK1/2 inducible proteins Jun b proto-oncogene (JunB) mediates ERK1/2 activation *via* the AP-1 complex, which increases the expression of several Th2 cytokines and drives Th2 cell differentiation, causing childhood asthma (Alam and Gorska, 2011). Besides, sprouty-2, a cytosolic adapter protein, also regulates receptor-mediated ERK1/2 activation by preventing c-Cbl-mediated degradation of EGF receptor, which also stimulates Th2 cell differentiation to amplify Th2 inflammation (Liu et al., 2008; Alam and Gorska, 2011; Sripada et al., 2021). However, further mechanism about ERK1/2 in childhood asthma is needed to explore.

**FIGURE 3:**
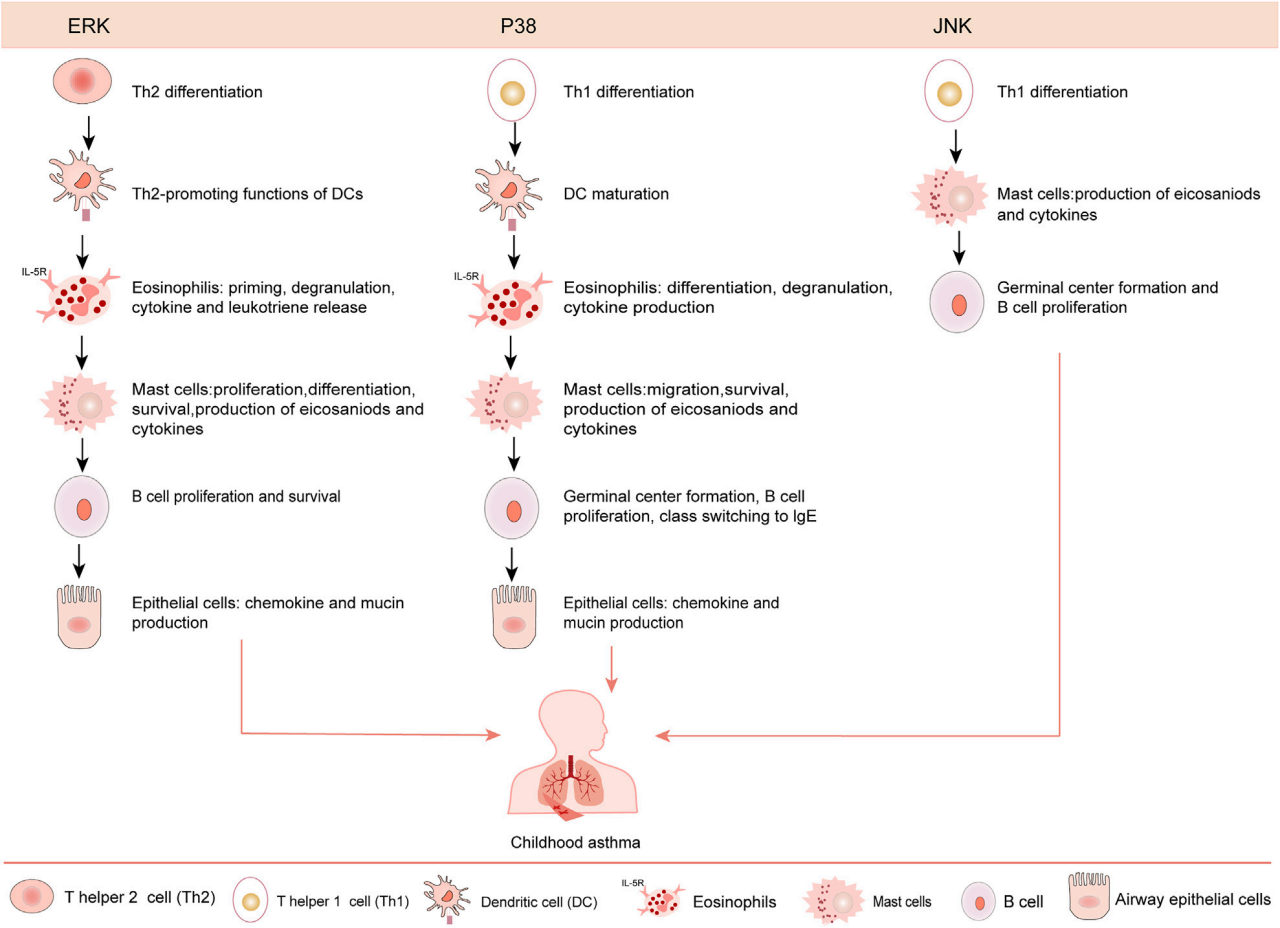
Roles of various MAPK signaling pathways in asthmatic pathogenesis. ERK favors Th2 cell differentiation, eosinophils priming, degranulation, cytokine and leukotriene production, mast cells proliferation/differentiation. The p38 MAPKs and JNK pathways regulate Th1 differentiation. The p38 MAPK contributes to eosinophil degranulation, migration and cytokine production and mast cell migration. The JNK participates in cytokine production by mast cells, regulates the proliferation of B cell, and exerts function in asthmatic pathogenesis.

In contrast to ERK1/2, p38 MAPKs and the JNK pathways favor Th1 differentiation. P38 MAPK and JNK activation contributes to the inflammatory response in asthma. Moreover, p38 MAPK is observed in the basal layer of the columnar epithelium, alveolar macrophage and bronchial epithelial, ect. driving basal metabolic processes for these cell type. That’s to say, p38 MAPK is vital for allergen induced epithelial production of IL-25 and thymic stromal lymphopoietin (TSLP), further mediating the type-2 allergic response in asthma (Yu et al., 2010; Lin et al., 2015; Southworth et al., 2018). Previous studies have confirmed that dual-specificity phosphatase1 (DUSP1) also plays vital role in anti-inflammation by deactivating MAPKs through dephosphorylation (Pulido and Lang, 2019; Xin et al., 2021; Theodorou et al., 2022; Xing and Wong, 2022). Besides, Studies have confirmed that JNK is essential for airway inflammation *via* modulating RAGE/β-catenin signaling (Huang et al., 2021a). Regretfully, further mechanisms between JNK and RAGE/β-catenin is deserved exploring (Figure 3).

### Genetic Susceptibility of Childhood Asthma

Although the immune mechanisms and signaling pathways of asthma are widely reported, complex interactions between genetic susceptibility and environmental influences also lead to childhood asthma (Thomsen, 2015). Studies have shown that the genetic susceptibility of asthma can be as high as 60–70%, which suggests that understanding the genetic basis of childhood asthma might unravel mechanisms, directing the treatment of asthma. So further genome-wide association studies (GWAS) is imminent due to the complexity and heterogeneity of asthma (Alizadeh et al., 2017; Kabesch and Tost, 2020). Consequently, it has been found that the main genetic risk factor for asthma is single nucleotide polymorphism (SNPs), which is a single-base pair, occurring on average one in 300 nucleotides (Lee et al., 2015). Asthma-associated SNPs in this locus are related to levels of mRNA expression of ORMDL3 in lymphoblastic cell lines using eQTL mapping, which associates SNPs with gene expression (El-Husseini et al., 2020). Non-synonymous SNP of The IL7R (rs6897932) and IL2RB (rs2284033) (Arenas-Ramirez et al., 2015) regulate asthma by regulating type II inflammation *via* activating three pathways: JAK-STAT, PI3K-Akt-mTOR (Patel and Chang, 2012), and MEK-ERK (El-Husseini et al., 2020). Approximately 88% of the disease-associated variants acquired from genome-wide association studies (GWASs) reside in non-coding regions (El-Husseini et al., 2020). Examples of pathways or networks that are implicated by GWASs in asthma are the IL33-IL1-RL1 receptor pathway, leading to eosinophilia, and the T-helper-2 (Th2) cytokine IL-5 and IL-4RA receptor, leading to eosinophilia and type 2 inflammation or viral response (CDHR3, ORMDL3^)^ (Zhang et al., 2019a; Basnet et al., 2019). For example, genetic variation at chromosome 17q12-21 is associated with childhood asthma but not adult asthma (Ferreira et al., 2019; Pividori et al., 2019). Subsequent investigations also linked SNPs at this locus to GSDMA, GSDMB, CRKRS, ZBPB2, and IKZF2 expression in whole blood cells and lung tissue.

## NcRNAs in the Regulation of Childhood Asthma

NcRNAs are a class of RNA transcripts that do not encode proteins, but they have been implicated in regulating gene expression at the epigenetic, transcriptional and post-transcriptional levels and affect the biological functions such as immune response, tissue repair and remodeling (Cech and Steitz, 2014; Cai et al., 2015; Xie and Liu, 2015; Zhang et al., 2019b). Mounting studies have shown that ncRNAs are involved in the occurrence and progression of childhood asthma. In this review, we mainly focus on the regulatory role of ncRNAs in the pathogenesis of childhood asthma and discuss the feasibility of ncRNAs as new biomarkers for the treatment of childhood asthma (Fasanaro et al., 2015) (Table 1).

**TABLE 1:** Classification of ncRNAs.

Type	Full name	Abbreviation	Size	Function
Housekeeper ncRNAs	Ribosomal RNA	rRNA	120–4,500 nt	Used as a scaffold for mRNA molecules to achieve protein synthesis
	Transfer RNA	tRNA	76–90 nt	Transfer of activated amino acids involved in protein biosynthesis
	Small nuclear RNA	snRNA	100–300 nt	Binding with protein factors to form nuclei karyon glycoprotein particles to perform splicing mRNA function
	Small nucleolar RNA	snoRNA	60–400 nt	rRNA of modification
Regulatory ncRNAs	MicroRNA	miRNA	21–23 nt	By complementary pairing of bases with the 3'-terminal untranslated region of the target mRNA
	Long non-coding RNA	lncRNA	>200 nt	Dose compensation effect, epigenetic regulation, cell cycle regulation and cell differentiation regulation
	Small interfering RNA	siRNA	20–25 nt	—
	Circular RNA	circRNA	100–10,000 nt	—

### Long Non-Coding RNAs in the Regulation of Childhood Asthma

LncRNAs, with a length of over 200 nt, play critical roles in regulating gene expression at multiple levels, including transcriptional, post-transcriptional levels, microRNA chelation and translation efficiency regulation and more (Hao and Zan, 2021; Wang et al., 2021). Although many studies have proved that lncRNAs and childhood asthma are inextricably linked (Wang et al., 2021), the roles of lncRNAs in childhood asthma remain unclear.

Recently, it has been reported that lncRNAs participate in regulating airway inflammation and remodeling (Ezegbunam and Foronjy, 2018; Liu et al., 2019a; Li et al., 2020a), and are helpful to further determine the biomarkers and therapeutic target of childhood asthma. Increasing numbers of studies suggest that lncRNAs affect the regulation of immune response, airway inflammation and cytokine expression (Dai et al., 2021). Moreover, lncRNAs also participate in the regulation of T helper (Th)1/Th2 imbalance, T regulatory (Treg)/Th17 imbalance, eosinophils dysfunction, macrophage polarization, airway smooth muscle cells (ASMCs) proliferation and so on (Zhu et al., 2020), to mediate childhood asthma.

Studies have found that LNC-000127 was not only closely related to Th2 inflammation but also positively regulated eosinophilic asthma. Malat1 has capability to modulate Th1/Th2 balance in asthma *via* MALAT1/miR-155/CTLA-4 axis. Therefore, MALAT1 and LNC-000127 could be used as a biomarker for eosinophilic asthma (Zhu et al., 2019). Additionally, Treg/Th17 imbalance was also associated with childhood asthma through neutrophil recruitment and exacerbation of airway inflammation, which was mediated by upregulating Th17-type cytokines (IL-17A, IL-17F) and downregulating Treg-type cytokines (IL-10, transforming growth factor (TGF-β) and so on (Westfall et al., 2021). LncRNA MEG3 regulated RORgammat expression by competitively sponge miR-17 and ultimately affected the balance of Treg/Th17 (Qiu et al., 2019; Hao and Zan, 2021; Wang et al., 2021). In addition, lncRNA RP11-401.2 upregulated in eosinophils dysfunction regulated eosinophilic asthma (Tian et al., 2018). Lnc-BAZ2B promoted M2 macrophage activation and was significantly upregulated in childhood asthma, whereas LncRNA PTPRE-AS1 was downregulated in macrophage polarization which mediated type II inflammation (Zeng et al., 2019). Regretfully, the detailed regulated mechanism of lncRNAs in childhood asthma is unclear. Besides, microarray analysis of CD4^+^ T cells of asthma patients showed that lncRNA ENST00000583179, lncRNA ENST00000579468 and lncRNA ENST00000444682 were positively correlated with the expression of IL-5 and IL-13, while lncRNA ENST00000583179 was positively correlated with the expression of IL-4 and IL-6. Interestingly, lncRNAs also regulated the expression of cytokines (IL-5 and IL-13), transcription factors (STAT5 and STAT6) and chemokines (CCL17 and CCL22), which mediated Th1 and Th2 inflammatory response in asthma. Nevertheless, its role in the pathogenesis of asthma remains to be further studied (Wang et al., 2017). Recently, lncRNAs SNHG8 and LINC01559 have been found to be directly involved in the occurrence and progression of childhood asthma through PI3K/AKT signaling pathway (Hao and Zan, 2021), and may serve as candidates for therapeutic strategies for childhood asthma (Table 2). Further elucidating the role of various lncRNAs in childhood asthma will help to understand the pathogenesis of asthma and develop potential treatment targets.

**TABLE 2:** Dysregulated lncRNAs in asthma.

LncRNAs	Targets	Expression	Mechanism	Signaling pathways	Clinical effects	Ref
Malat1	miR-155, miR-150	Upregulation ↑	Sponge miR-155 and hinder its bond with CTLA-4 to break Th1/Th2 balance, act as a ceRNA for miR-150, induce ASMCs proliferation	MALAT1/miR-155/CTLA-4 axis, eIF4E/Akt signaling	Act as markers in dysregulated Th1/Th2 imbalance, modulate airway remodeling in asthma	(Lin et al., 2019; Liang and Tang, 2020)
LNC-000127	—	Upregulation ↑	Promote Th2 inflammation in eosinophilic asthma	TCR/STAT/GATA3 pathway	Distinguish the phenotype of eosinophilic asthma	Zhu et al. (2019)
lncRNA MEG3	miR-17	Upregulation ↑	Repress the bond of miR-17 to RORgammat to prevent RORgammat, mRNA degradation	miR-17/RORgammat	Regulate the balance of Treg/Th17	Qiu et al. (2019)
lncRNA RP11-401.2	—	Upregulation ↑	Promote eosinophils dysfunction	—	Regulate eosinophilic asthma	Tian et al. (2018)
lnc-BAZ2B	BAZ2B	Upregulation ↑	Increase BAZ2B to enhance IRF4 and M2 macrophage activation	—	A target for modulating type 2 inflammation	Xia et al. (2021)
ENST00000444682	SMAD7, WNT2B, C/EBP, T-bet, NF-κB	Upregulation ↑	Positively correlated with IL-13 and IL-5, negatively correlated with IL-6	SMAD7 cAMP/C/EBP/T-bet/NF-κB	Modulate Th2 cell differentiation and the related proinflammatory factor production	Qiu et al. (2018)
ENST00000566098		Upregulation ↑	Positively correlated with IL-13			
ENST00000583179		Upregulation ↑	positively correlated with IL-13, negatively correlated with IL-4 and IL-6			
ENST00000579468		downregulation ↓	Positively correlated with IL-5 and IL-13			
PVT1	c-MYC	Upregulation ↑ (severe asthma) Downregulation ↓ (non-severe asthma)	DEX increases c-MYC, which can bind to LncRNA PVT1 to promote ASMCs proliferation in severe asthma.the knockdown of LncRNA PVT1 reverses the inhibitory effect of DEX, amplifying glucocorticoid insensitiveness	PVT1/c-MYC	Distinguish ASMCs phenotype and regulate glucocorticoid sensitiveness	Austin et al. (2017)
lncTCF7	TIMMDC1/AKT	Upregulation ↑	Enhance ASMCs growth and migration *via* activating the TIMMDC1/AKT signaling pathway	TIMMDC1/AKT pathway	A potential therapeutic for airway remodeling	Fan et al. (2019)
lncRNA GAS5	miR-10a/BDNF	Upregulation ↑	Promote the proliferation of ASMCs	miR-10a/BDNF signaling pathway	Proliferation of ASMCs	
LINC00882	miR-3619-5p	Upregulation ↑	sponge miR-3619-5p and prevent its bond to b-catenin to enhance PDGF-induced fetal ASMCs proliferation	Wnt/β-catenin signaling	Modulate ASMCs proliferation in pediatric asthma	Liu et al. (2019b)
CASC7	miR-21	Downregulation ↓	Sponge miR-21 and suppress its bond to PTEN, thus enhancing PTEN expression (elevate corticosteroid sensitivity)	PI3K/AKT signaling pathway	Enhance glucocorticoid sensitivity	Liu et al. (2020)
LncRNA SNHG8 LINC01559		Upregulation ↑	ECM-receptor interaction, focal adhesion, beta-alanine metabolism	PI3K/AKT signaling pathway	Regulate cell proliferation, migration	

In sum, lncRNAs associated with cytokines, Th2-related transcription factors (STAT5 and STAT6) and Th2 related chemokines (CCL17 and CCL22) to affect the balance of Th1/Th2 (Wang et al., 2017). In addition, lncRNAs may act as regulator of Th1 and Th2 inflammation in the pathogenesis of asthma through lncRNA-miRNA-mRNA axis. For example, lncRNA fantom3-9230106C11 binded to miRNAs and transcription factors to mediate Th2 cell differentiation and regulated Th2 inflammation (Wang et al., 2019). However, only a small number of lncRNAs were applied to the clinical diagnosis and treatment of childhood asthma. Further research associated with lncRNAs is needed in the field of asthma therapy.

### MicroRNAs Act on Target Pathways and Regulatory Mechanisms of Childhood Asthma

MiRNAs are a class of small ncRNAs (approximately 21–25 nucleotides) that regulate gene expression and cellular function by primarily bind to the 3′ untranslated region (3′ UTR) of mRNAs (Simpson et al., 2014; Kho et al., 2018). MiRNAs play vital roles in regulating Th2 activation, differentiation and proliferation by directly or indirectly acting on target genes (Rebane and Akdis, 2013; Qiu et al., 2018). On one hand, miRNAs regulate airway inflammation of childhood asthma by increasing Th2 cytokine secretion to decrease Th1 cytokine secretion and promote the differentiation of CD4^+^ T cells into Th2 (Midyat et al., 2016; Aripova et al., 2020). On the other hand, miRNAs also play roles in hyperplasia and hypertrophy of airway smooth muscle cells (Specjalski and Niedoszytko, 2020).

MiRNAs are involved in the pathogenesis of asthma by regulating inflammatory reaction. Specifically speaking, a core set of miRNAs were involved in childhood asthma including the downregulated let-7 family and upregulated miR-155, miR-21, miR-142-5p, miR-142-3p, miR-223, and miR-146a/b, etc. (Specjalski and Niedoszytko, 2020; van den Berge and Tasena, 2019). Let-7 microRNAs belongs to a family of highly conserved microRNA and comprises the most abundant miRNAs in lungs (Karam and Abd Elrahman, 2019; Cañas et al., 2020). Kumar et al. (2014) showed that let-7 family members were decreased in ovalbumin-sensitized animal models (Huang et al., 2020; Weidner et al., 2021), playing a proinflammatory role in asthma. Furthermore, let-7 family inhibited the secretion of IL-13 by directly targeting IL-13 transcript (Huang et al., 2020; Xu et al., 2020). Among numerous miRNAs reviewed in this field, miR-146, miR-155, and miR-223 have been identified as inflammatory response miRNAs that are up-regulated by NF-κB (Boldin and Baltimore, 2012; Kumar et al., 2014). MiRNA-21 is involved in the pathogenesis of asthma by limiting the activation of IL-12/IFN-γ and the differentiation of Th1 and Th2. It has been described that lack of miR-21 in mice may cause the increasion of levels of IFN-γ secreted by Th1 cells and the decreasion of pulmonary eosinophils, hence inhibiting the inflammation (Das et al., 2014).

MiRNAs are also involved in airway remodeling in asthmatic mice model. Ras homolog family member A (RhoA) of the Rho family GTPases regulated airway remodeling through regulating mesenchymal stem cell (MSC) differentiation. MiR-133a negatively regulated RhoA expression and bronchial smooth muscle cells (BSMCs) contraction (Chiba, 2020) to influence airway remodeling. Moreover, miR-26a induced human airway smooth muscle cells (HASMCs) hypertrophy (Mohamed et al., 2010). MiR-10a reduced mitogen-induced HASMCs proliferation (Hu et al., 2014) to regulate airway remodeling.

MiRNAs are not only the regulators in asthma pathogenesis, but also the targets of asthma therapeutics. Further studies illustrated miRNAs inhibited asthma through downregulation or antagonism of disease-related miRNA (Wang, 2010). Growing evidence showed that up-regulated miRNAs could be inhibited by miRNAs inhibitors or synthetic miRNAs oligonucleotides against miRNAs activity, and the administration of miRNAs inducers that increased tissue-specific miRNAs expression might be another treatment for asthma (Lukiw, 2013; He et al., 2014). For example, miR-155 knockout and miR-106a knockdown alleviated asthma though diminishing airway inflammation, mucus hypersecretion and airway Th2 cytokine levels (Mattes et al., 2009; Huang et al., 2014; Malmhäll et al., 2014). MiRNA-221 blockade suppressed airway inflammation. Moreover, Let-7 miRNA inhibition reduced airway hyperresponsiveness and subepithelial fibrosis by decreasing IL-13 levels to suppress airway inflammation and attenuate mucus metaplasia (Chiba et al., 2009; Kumar et al., 2011) (Table 3).

**TABLE 3:** Roles of selected miRNAs in childhood asthma.

MiRNAs	Targets	Expression	Functions	Ref
Let-7 family	—	Downregulation ↓	IL-13 downregulation	(Karam and Abd Elrahman, 2019; Huang et al., 2020)
miR-155	IL-4,IL-5, IL-13, IL-17a,CTLA-4, CD4^+^ T	Upregulation ↑	Enhanced inflammation and mucus secretion regulation of T-cell activation influence on proliferative response	(Zhou et al., 2016a; Zhang et al., 2017)
miR-210	—		Inhibition of Treg function	Long et al. (2016)
miR-181a	—		Augmenting sensitivity of T cells to peptide antigens	
miR-21, miR-19a	—	Upregulation ↑	Promoting differentiation of T cells towards Th2	(Sawant et al., 2013; Long et al., 2016)
miR-221-3p	PTEN	Upregulation ↑	IL-4 upregulation	Zhou et al. (2016b)
miR-1248		Upregulation ↑	IL-5 upregulation	
miR-146a/b, miR-28-5p		Upregulation ↑	IL-5 inhibition downregulate miR-146a/b and miR-28-5p expression and activate CD8^+^ T cells	(Tsitsiou et al., 2012; Yang et al., 2017)
miR-323-3p, miR-181a, miR-26a	SMAD2, SMAD3	Upregulation ↑	TGF-β-dependent signaling pathway modulation	Kärner et al. (2017)
miR-513-5p, miR-22-3p, miR-625-5p		Upregulation ↑	Inhibition of Th1 cytokines including IL-12, and interferon-γ	Dong et al. (2016)
miR-221, miR-485-3p			Downregulate Spred-2	Liu et al. (2012)
miR-1	—	Downregulation ↓	Inhibits the secretion of IL-4, -5, -8, TNF-α; regulates Th1/Th2 balance	Tian et al. (2018)
miR-218-5p	CTNND2	Downregulation ↓	Inhibits bronchial hyperresponsiveness, eosinophilic airway inflammation	Liang et al. (2020)

### CircRNAs in the Regulation of Childhood Asthma

CircRNAs are a special class of ncRNAs that function as miRNA sponges to indirectly regulate downstream mRNA expression and epigenetically influence various biological processes, especially in cancers (Zhao et al., 2019; Huang et al., 2021b). However, the contribution of circRNAs to childhood asthma progression remains unknown. In recent years, circRNAs have attracted extensive attention in the pathogenesis of childhood asthma.

Huang et al. (2019) showed that circ-0005519 could regulate the secretion of IL-13/IL-6 by competitively sponging let-7a-5p in CD4^+^ T cells from asthma. Previous studies also found that circ-0002594 was a proinflammatory factor in Th2-mediated asthma (Huang et al., 2021b). Circ-0000723 could sponge miR-214 to impact the balance of Th1/Th2 by runt-related transcription factor (RUNX) signal transduction. Moreover, circRNAs also play vital roles in airway smooth muscle cells (ASMCs). Mounting evidence showed that circERBB2 and circHIPK3 could sponge miR-98-5p and miR-326, respectively, and promoted the proliferation of ASMCs by targeting IGF1R and STIM1 (Lin et al., 2020; Huang et al., 2021c). Circ-0001359 could attenuate airway remodeling by targeting FoxO1-dependent M2-like macrophage activation, with sponging miR-183-5p (Shang et al., 2020; Mathis et al., 2021) (Table 4). At present, although there are many studies on the competitively sponging of circRNAs to miRNAs, the specific effect of these circRNAs on asthma is not completely clear. So, the pathogenesis of circRNAs in childhood asthma should be further studied in order to seek for potential diagnostic and therapeutic targets of childhood asthma, which can be used as a new direction of targeted drug therapy.

**TABLE 4:** Roles of circRNAs in childhood asthma.

CircRNAs	MiRNA sponges	Species/Cells	Targets	Functions	Ref
circ-0005519	Let-7a-5p	Human/CD4^+^ T cell	IL-13 IL-6	IL-13 and IL-6 expression	Huang et al., 2019; Wang et al., 2021)
circ-0002594	miR-16-5p, -503-5p, -514a-3p, -587, and let-7e-5p	Human/CD4^+^ T cell	—	Related to Th2-mediated asthma	Huang et al. (2021b)
circ-0000723	miR-214	Mice/CD4^+^ T cell	RUNX	Th1/Th2 balance	Qiu et al. (2017)
circERBB2	miR-98-5p	Human/ASMC	IGF1R	proliferation in ASMCs	Huang et al. (2021c)
circ-0001359	miR-183-5p	Mice/macrophage	FoxO1	M2 macrophage activation	Shang et al. (2020)
circHIPK3	miR-326	Human/ASMC	STIM1	The proliferation, migration and apoptosis in ASMCs	Lin et al. (2020)

### NcRNAs Function as Targets for Asthma Diagnosis and Treatment

Although high-dose inhaled corticosteroids and long-acting β2 agonists have been improved for the treatment of asthma, none of these treatments have been shown to alter the natural history of the asthma, and some patients are still failing in these treatments (Sweeney et al., 2012). NcRNAs play an essential role in the treatment and prognosis of asthma. Hence, asthma has been turned to lncRNAs, miRNAs and circRNAs in search of new breakthroughs (Zampetaki et al., 2012; Milagro et al., 2013). Evidences have implicited that lncRNAs, miRNAs and circRNAs can be used as biomarkers of sensitivity and early diagnosis of asthma (Zampetaki et al., 2012). For instance, the expression of lncRNA CASC2 in serum was at a lower level in asthma children than healthy individuals, which suggested that lncRNA CASC2 might be involved in childhood asthma (Yang et al., 2022). Moreover, lnc-BAZ2B also played a crucial role in exacerbating the progression of childhood asthma (Xia et al., 2021). It can be inferred that lncRNA CASC2 and lnc-BAZ2B may serve as potential diagnostic biomarkers, and are expected to become new targets for childhood asthma treatment in the near future. Besides, many surveys have identified that miRNAs such as let-7a, miR-146b-5p, miR-21, miR-532-5p, miR-155 and so on, are promising to be used as diagnostic biomarkers and therapeutic targets in childhood asthma (Kho et al., 2018; Li et al., 2020b; Xu et al., 2020). CircRNAs also play crucial roles to regulate childhood asthma through circRNA-miRNA-mRNA regulatory network and can be served as potential biomarkers and therapeutic targets in childhood asthma (Chen et al., 2021; Wang et al., 2022). Although siRNAs are one of the regulatory ncRNAs, seldom studies have demonstrated the roles of siRNAs in the pathogenesis of childhood asthma. Recently, only several studies have implicated the therapeutic effects of synthetic siRNA in allergen-induced asthma models (Miyamoto et al., 2011; Chen et al., 2017). Consequently, research of drugs related to ncRNAs, especially lncRNAs, miRNAs and circRNAs, may become a new direction in the field of targeted asthma therapy.

## Conclusion and Perspective

In this review, we mainly discuss childhood asthma. Children are special categories of patients and the symptoms of many other diseases are similar with asthma, which makes the diagnosis of asthma difficult. This review provides a novel strategy to diagnose and treat childhood asthma by targeting ncRNAs. Despite the treatment of asthma by inhaling corticosteroids, long acting β agonists, and leukotriene modifiers could reduce symptoms, the burden of asthma remains high. As far as childhood asthma, there is no evidence that early treatment decreases the risk of subsequent asthma or alters its natural history (Depner et al., 2014; Ducharme et al., 2014). It is widely quoted that 5–10% of the asthmatic population have severe asthma, suffering a significant health and socioeconomic burden (Papi et al., 2018; Agache et al., 2021). Facing these challenges, biomarker-directed therapy is more and more attractive and biomarkers for asthma have potential utility for distinguishing the inflammatory endotype, predicting responsiveness to specific treatments, monitoring success of a selected treatment option, and assessing the risk of disease progression (Agache and Rogozea, 2017; Cosmi et al., 2017). Accordingly, the discovery of lncRNAs, miRNAs and circRNAs offers a new opportunity for understanding the pathogenesis of childhood asthma (Rundell et al., 2015) and it is necessary to develop ncRNAs as new therapeutic targets for asthma.

Clinically, we still lack effective treatment measures for refractory asthma. NcRNAs have been considered to be one of the most promising and novel therapeutic targets for childhood asthma. Correctly, lncRNAs contributing to the regulation of airway remodeling and glucocorticoid sensitivity during transcription make it a potential biomarker for the preclinical identification, diagnosis, prognosis, phenotypes of asthma as well as therapeutic targets. However, there is no research to discuss the relationship between lncRNAs and Th2 cell and T follicular helper cells, which might be the focus of future research. In the same way, miRNAs also have multiple potential targets that may coordinate or antagonize each other’s functions. Restoring normal physiological levels of miRNAs in asthma, such as miRNA mimics, inhibitors, might have the potential to improve clinical outcomes (Ameis et al., 2017). Actually, ncRNAs still exist limitations, for example, how to accurately locate at a specific target or a certain organ, and whether through specific chemical modification of nucleic acid drugs or not, possible off-target effects of nucleic acid drug, etc. which is still worth exploring. Besides, there are still some unexpected side effects on ncRNAs, such as the disruption of the immune response and incompleteness during the treatment because of individual differences and so on. Luckily, if given appropriate immunotherapy and individualized treatment, these side effects may be reduced.

Nevertheless, due to complicated crosstalks between ncRNAs and inflammatory pathways in asthma, the expression of single lncRNAs, miRNAs and circRNAs may not be a truly reliable biomarker. Thus, several attempts have been made in finding asthma associated with pathways to provide a new avenue to treat asthma. For example, the metastatic-associated lung adenocarcinoma transcript 1 (MALAT1)/miR-155/CTLA-4 axis has the ability to regulate Th1/Th2 balance in asthma. MALAT1 is upregulated in the blood of asthmatic patients, while the miR-155 is downregulated, and the Th1/Th2 ratio is decreased, which suggests that Th1 inflammation is weakened and Th2 inflammation is amplified, so it can be used as a marker of inflammatory disorders in asthma (Liang and Tang, 2020). Thus, future studies should be more focused on human settings. Application of lncRNAs, miRNAs and circRNAs as non-invasive biomarkers should be investigated with an emphasis of possible determination of disease endotype and predicting treatment effects. Recently, the roles of all ncRNAs in the pathogenesis of childhood asthma have not been clarified, which should be further studied in order to seek for potential diagnostic and therapeutic targets of childhood asthma.
